# Current Developments in µMAS NMR Analysis for Metabolomics

**DOI:** 10.3390/metabo9020029

**Published:** 2019-02-06

**Authors:** Covadonga Lucas-Torres, Alan Wong

**Affiliations:** NIMBE, CEA, CNRS, Université Paris-Saclay, CEA Saclay, 91191 Gif-sur-Yvette, France; covadonga.lucas-torres-perez@cea.fr

**Keywords:** NMR, metabolomics, magic-angle spinning, HR-MAS, micro-MAS, metabolic profiling

## Abstract

Analysis of microscopic specimens has emerged as a useful analytical application in metabolomics because of its capacity for characterizing a highly homogenous sample with a specific interest. The undeviating analysis helps to unfold the hidden activities in a bulk specimen and contributes to the understanding of the fundamental metabolisms in life. In NMR spectroscopy, micro(µ)-probe technology is well-established and -adopted to the microscopic level of biofluids. However, this is quite the contrary with specimens such as tissue, cell and organism. This is due to the substantial difficulty of developing a sufficient µ-size magic-angle spinning (MAS) probe for sub-milligram specimens with the capability of high-quality data acquisition. It was not until 2012; a µMAS probe had emerged and shown promises to µg analysis; since, a continuous advancement has been made striving for the possibility of µMAS to be an effective NMR spectroscopic analysis. Herein, the mini-review highlights the progress of µMAS development—from an impossible scenario to an attainable solution—and describes a few demonstrative metabolic profiling studies. The review will also discuss the current challenges in µMAS NMR analysis and its potential to metabolomics.

## 1. Introduction

The study of metabolome within the organism, tissue, cell, fluid or other biomaterials can be carried out by several analytical techniques, NMR is one of the accomplished spectroscopic methods. It is capable of accurately annotating the metabolic profile with high reproducibility in a quantitative fashion. The versatility of NMR to different sampling morphologies (from liquid to semi-solid) and volume (from μL to nL) has significantly widened the application to metabolomics. Hence, it is utmost important that new strategies for NMR instrumentation and methodologies [1,2,3,4] must be continued striving forwards to preserve NMR spectroscopy as a frontline analytical platform in metabolomics.

NMR is intrinsically hindered by the low detection sensitivity, with an average detection of about 20–30 metabolites in the micromolar sample. As a result, NMR is often limited by the need for large sampling volume (ca. 500 μL), obstructing the analysis of microscopic level samples. The use of high-field magnets (i.e., B_0_ > 16 T) is an approach to enhance the sensitivity. However, the running cost is exponential with the field. A cost-effective method to microscopic sample is the use of miniature μ-size detection coil for maximizing the filling factor (i.e., the sample volume close to the detection volume). The prime concern and difficulty of using μcoil is maintaining the *high spectral resolution* data for metabolomics studies; this is generally done by matching the magnetic susceptibility of the µcoil materials [5], or by the use of zero-susceptibility µcoil [6]. The method of μcoil in solution NMR has now become a routine approach to study nL samples; and it has advanced to high-throughput capability [7] with the possibility of combining with liquid chromatography in metabolomics [8] and with advanced sorting microfluidics [9,10,11]. The fact that microscopic samples – organisms, tissues or cells – can be optimally detected and analyzed with a cost-effective approach makes μcoil an extremely appealing technology for μNMR spectroscopy.

Despite the advancements, μcoil in magic-angle spinning (MAS)—for complex samples like the intact organism, tissue and cell—is substantially lagging. This is due to the challenges of implementing a durable MAS stator with a μcoil that can endure the rapid sample spinning without sacrificing the quality (both resolution and sensitivity) of data acquisition. The early designs of μMAS were centering the idea of implanting an additional μcoil onto a conventional MAS probe and utilizing the existing stator to propel the sample spinning. The first μMAS was introduced in 2006 with a piggybacked design [12]. It consists of the μg-sample piggybacking on top of a standard rotor for sample spinning. Additional static μcoil is placed around the sample for detection with an optimal filling factor. Shortly after in 2007, another approach was introduced. It utilizes the concept of inductive coil coupling [13]. A self-inductive resonator (denoted as magic-angle coil spinning (MACS)) is wrapped around the μg-sample and placed inside a standard rotor. Essentially, both the MACS and the μg-sample are spun ensemble converting a conventional MAS probe to a μMAS by the inductive coupling between the MAS coil and the spinning MACS μcoil.

In 2012, JEOL Resonance Inc. introduced the first commercial standalone μMAS probe, in which the MAS stator consisted of a fixed 0.75-mm μcoil (coil-diameter) [14,15]; later in 2015, Bruker launched a 0.7-mm μMAS probe [16]. These commercial μ-probes are explicitly designed for ultra-fast sample spinning up to 110 kHz offering the possibility of acquiring narrow ^1^H NMR signals for characterizing solid materials. Since many new and exciting NMR applications have emerged in the field of material science [17] and biological science [18]. However, the optimal spectral resolution acquired from these μMAS probes is highly insufficient for metabolic investigations. It was not until 2012; a μMAS probe had emerged and demonstrated the possibility of metabolic profiling with μg tissues [19]. This mini-review highlights the different strategies and progress of the development of μMAS NMR spectroscopy towards μg-scale biospecimens.

### The Basic Criteria of µMAS for Metabolomics

As in most NMR-based metabolomic studies, to assure reliable dataset are acquired for the analysis, the method—μMAS NMR in this case—must at minimal attain the following essential specification criteria:

- Detection sensitivity: The unequivocal identification and quantification of the metabolites are the critical aspects of metabolomics. In NMR, the adequate signal-to-noise ratio (SNR) is essential; the standardized requirement for the limit of detection (LOD) and quantification (LOQ) are SNR ≥ 3 and 10, respectively [20,21].
- Spectral resolution: Aside from the fact that high spectral resolution improves sensitivity, it also facilitates the unfolding of the dense metabolic NMR signal patterns (e.g., the 3–5 ppm range in a ^1^H spectrum) and allowing thorough peak analyses (identification and quantification). The acceptable ^1^H resolution by HR-MAS NMR is about 0.005 ppm (i.e., 2.5 Hz at 11.7 T) [22].
- Spectral repeatability: Metabolic profiling relies predominantly on multivariate and quantification data analysis; thus, the capability of acquiring homogenous data from the same sampling pool is crucial. This depends on the stability of the NMR instrumentation (including the probe) and also the sample preparation.
- Sample preparation: The precision and accuracy in the sample obtainment and sample preparation are of importance to avoid biased interpretation of the metabolic responses; therefore, a clean and reliable sample preparation must be established for all study models.

These criteria are essentially the basic guidelines for the development. In the search for a suitable µMAS technology, several approaches have been attempted; and among them, HR-MACS and HR-µMAS show promising signs for metabolic investigation with a capability for acquiring good quality data for an unbiased data analysis and interpretation.

## 2. µMAS Approaches to Metabolomics

### 2.1. µMAS Using A Sample Configuration

**Figure 1:**
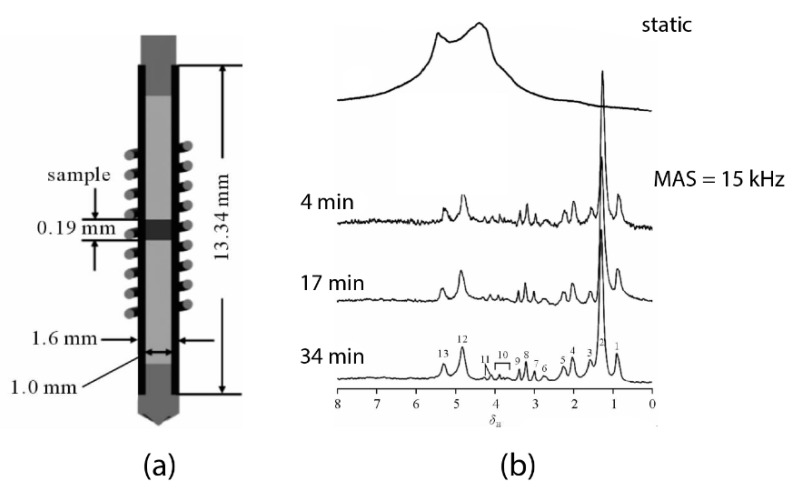
(**a**) The sample-configuration in a 1.6-mm rotor with Kel-F inserts [23]. (**b**) Spectral comparison ^1^H magic-angle spinning (MAS) NMR of a mouse muscular tissue at 21.1 T (900 MHz for ^1^H) between non-sample spinning acquisition and spinning at 15,000 Hz with increasing acquisition time [23]. Modified with permission from the author.

Efforts have been made to analyze mass-limited samples with an improved spectral quality. The conventional methods are by: (i) applying high magnetic field acquisition with a standard volume NMR probe, or by (ii) the use of diluted NMR-observable nuclei to limit the internal spin-spin interaction. In 2013, an attempt was made using a standalone 1.6-mm MAS probe to analyze a 150 μg skeletal muscle tissue [23] at 21.1 T (900 MHz for ^1^H). To maximize the spectral quality acquisition (in both sensitivity and resolution), the μg biopsy is strategically placed at the center of the rotor by two susceptibility-matched Kel-F inserts (Figure 1a) for facilitating the field shimming. Moreover, the inserts also prevent from sample leakage during the rapid spinning. Indeed, the high magnetic field acquisition permits an excellent sensitivity for μg tissues (SNR = 37 in just 4-min acquisitions). However, as shown in Figure 1b the resolution is highly insufficient for metabolic profiling; aside from the significant lipid signals, only a few metabolites (i.e., glutamine, creatine/lysine, choline, and sugar derivatives) can be considered identifiable from the spectrum.

This substandard resolution is ascribed to the susceptibility broadening (ΔS ∝ $B_{o}\Delta \chi_{\mathrm{ij}}/r_{\mathrm{ij}}^{3}$), with the presence of large magnetic susceptibility gradients (Δχ_ij_) between the μg tissue and the non-spinning components of the MAS stator including the wire of the µcoil (i.e., copper-wire). The submillimeter apart (*r_ij_*) between the sample and stator renders a substantial susceptibility broadening. Also, the study relied on a high magnetic field *B_o_* (21.1 T) for increasing the sensitivity with a μg tissue, but it affiliated at the cost of susceptibility broadening that could obscure the J-splitting for metabolic identifications. 

### 2.2. µMAS Using a Spinning µCoil: High-Resolution Magic-Angle Coil Spinning (HR-MACS)

**Figure 2:**
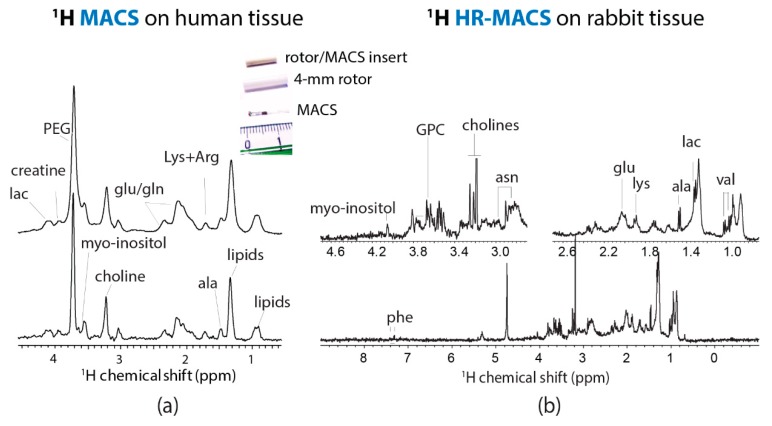
(**a**) ^1^H MACS NMR of approximately 500 µg of human tissue at 400 MHz with MAS at 1360 Hz, with about 15 identified metabolites. Adapted with permission from [19]. Copyright (2012) American Chemical Society. (**b**) ^1^H HR-MACS NMR of 250 µg rabbit kidney tissue at 500 MHz with MAS at 300 Hz. The improved spectral resolution offers a rich metabolic profile with about 20 identified metabolites. Adapted with permission from [24]. Copyright (2012) American Chemical Society.

The design of HR-MACS [24] is originated from MACS [13]. It is a straightforward approach for converting a standard MAS probe into a µMAS by using an inductive-coupled spinning µcoil. Essentially, a standard size rotor contains a small sampling capillary with a wound µcoil. The ensemble spinning at magic-angle suppresses the susceptibility line-broadening effect (ΔS ∝ $B_{o}\Delta \chi_{\mathrm{ij}}/r_{\mathrm{ij}}^{3}$) ascribed from the sample, capillary, and µcoil. The main advantage of MACS is its versatility; it can readily adapt to different NMR probes (liquid or MAS) at various fields. The teams of Wong and Nicholson have carried out an evaluation of the original MACS for metabolic profiling with 500 µg of animal and human tissues [19] (Figure 2a). Despite the seven-fold sensitivity enhancement by MACS, the spectral resolution is visibly poor (0.1 ppm at best, or 40 Hz at 9.4 T) as compared to that obtained from HR-MAS with mg tissues; however, a few important metabolites (i.e., lipids, lactate, choline, creatine) were identified. This report had offered the first glimpse of profiling µg tissues by µMAS NMR.

Unlike the aforementioned line-broadening, the broad line width ascribed from MACS is originated from the sample temperature gradient generated by the eddy current in a spinning µcoil, and the insuppressible (by MAS) anisotropic magnetic susceptibility of the rotor insert [24]. The design of HR-MACS is essentially a refinement of MACS by minimizing these effects to achieve high spectral quality data down to 0.02 ppm (10 Hz at 11.7 T). ^1^H HR-MACS NMR was first demonstrated on a 250 µg rabbit kidney tissue biopsy (Figure 2b). The resolution has dramatically improved from 0.10 to 0.02 ppm—with the visible signature doublets of alanine and valine—while with a 6.7-fold enhancement in sensitivity. To put in a perspective of the sensitivity gain, without HR-MACS (or µcoil in general), it would require 45 times (6.7^2^) longer in acquisition for a conventional 4-mm HR-MAS to obtain a spectral data of a 250 µg tissue with the same SNR. The resultant quality from HR-MACS has permitted rich-metabolic profiling with a total of 25 metabolites, on par with the HR-MAS NMR from an mg-scale tissue.

**Figure 3:**
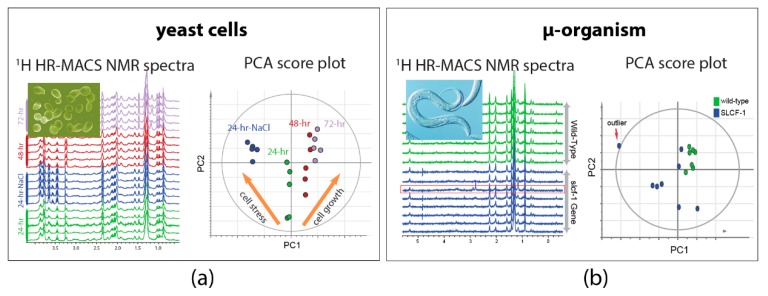
^1^H HR-MACS NMR spectral comparison and the principal component analysis (PCA) of (**a**) different yeast cells groups and (**b**) different strains of *C. Elegans*. Each one of the score plot corresponding to a PCA displays the quality data validated by the explained variation (R^2^X) and the predicted variation of the models (Q^2^). In (a) the PCA (with R^2^X(sum) = 0.793 and Q^2^(cum) = 0.737) clearly discriminates the different cell conditions (stress and growth). Adapted with permission from [25]. Copyright (2014) Frontiers in Chemistry. In (b) the PCA (with R^2^X(sum) = 0.964 and Q^2^(cum) = 0.8) confirms the reliability of the discrimination between the wild-type *C. Elegans* (green) and the strain of slcf-1 mutant (blue) with only 12 individuals. Adapted with permission from [26]. Copyright American Chemical Society.

Exploiting the high-quality acquisition with HR-MACS, ^1^H NMR-based metabolomics were performed on µg-samples of intact organisms. The demonstration on the intact yeast cells of *Saccharomyces cerevisiae* shows the capability of HR-MACS to comprehensively profile the metabolite constituents (with about 20 metabolites) in a 250 nL of cells, allowing to apply multivariate data analysis to precise their different conditions (Figure 3a) [25]. Another explorative study with HR-MACS was carried out with the whole µ-sized organism of *Caenorhabditis Elegans* (*C. elegans*) [26]. The resultant ^1^H HR-MACS spectral resolution, impressively, demonstrated to be better than that of HR-MAS, permitting identification of a metabolic profile with 31 metabolites from a sample with only about 12 worm individuals. This result represents the highest number of NMR-identified metabolites in a heterogeneous sample using µMAS. The high quality facilitates a discriminative analysis of two different strains of worms (wild-type *vs* slcf-1 mutant) (Figure 3b). These two demonstrative studies in Figure 3 not only illustrate the high-quality spectra obtained by HR-MACS but also highlight the possibility of acquiring data with modest repeatability for the multivariate data analysis.

Pushing the detection limit, HR-MACS has successfully detected metabolite signals from a single individual of *C. elegans* with the aid of a high magnetic field (18.8 T, 800 MHz for ^1^H) [26]. Although the resultant spectrum is inadequate for characterizing the metabolic activities due to the long acquisitions period (10 + h) and the presence of a substantial susceptibility broadening (ΔS ∝ B_0_), this result represents the first, and only, ^1^H MAS NMR detection of a single intact submillimeter organism.

One major flaw of HR-MACS is the µcoil itself: the strenuous process in fabrication and the fragility of the µcoil. Currently, the most effective approach for assembling an HR-MACS µcoil is by manually-winding, which is a labour-intensive process; the degradation of the NMR performance from the µcoil is inevitable due to the continuous centrifugation pressure exerted upon the µcoil. Therefore, with luck prevail the current-state of HR-MACS can only be applied to study with a small number of sampling. 

Several reports have acknowledged the difficulty in µcoil fabrication. A new design on-chip MACS [27] was introduced for adopting an automated fabrication with a robotic wirebonding technology. It enables rapid manufacturing of over 100 on-chip MACS in a single procedure; however, the reported spectral resolution was rather poor with about 1 ppm (500 Hz at 11.7 T) in line width. Another MACS design (monolithic MACS) was later introduced based on a 2D-printing technology [28]. Although the manufacturing efficiency was inferior to wirebonding, it offered a better spectral quality with a line width of 0.1 ppm (50 Hz at 11.7 T). 

### 2.3. A Standalone High-Resolution µMAS Probe (HR-µMAS)

**Figure 4:**
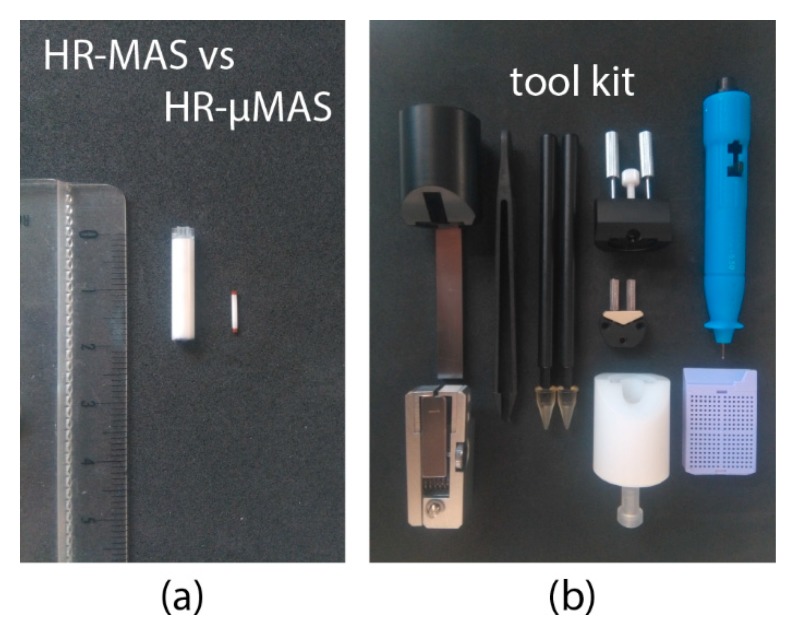
(**a**) MAS rotor comparison between 4-mm HR-MAS and 1-mm HR-µMAS. (**b**) A typical toolkit for the sample-preparation of an HR-µMAS experiment.

In 2015, a standalone high-resolution capable µMAS probe (denoted as HR-µMAS) was introduced [29]. It has the capacity of acquiring high-quality data from a 500 nL sample, about a 50-fold smaller than that from a conventional HR-MAS (Figure 4a). It was achieved by an extensive modification on a standard 1-mm µMAS probe by minimizing the susceptibility gradient (Δχ_ij_). Practically, the material of the MAS stator and the coil-wire were replaced with susceptibility-matched materials. The resultant resolution from HR-µMAS offers a ten-fold improvement with 0.01 ppm (5 Hz at 11.7 T) (as compared to that in Figure 1b). Unlike the previous HR-MACS design, HR-µMAS is a robust probe with a secured µcoil offering the possibility of large-scale studies (i.e., large sampling-replicates). Moreover, homo- and hetero-nuclear correlation experiments are feasible with HR-µMAS and have been demonstrated on ^1^H-^1^H TOCSY and ^1^H-^13^C HMQC experiments [29]. However, one must be cautious with the detection sensitivity.

**Figure 5:**
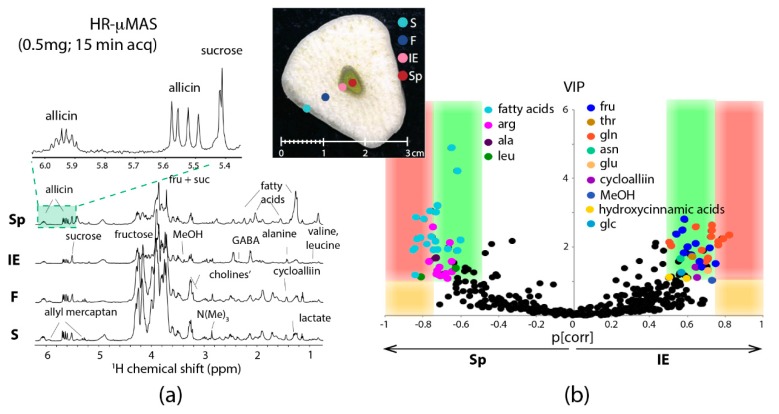
(**a**) ^1^H HR-μMAS NMR spectra of the four regions of a garlic clove tissue at 500 MHz spinning at 4000 Hz. The inserted photo displays the four different anatomical regions: S skin, F flesh, IE inner epidermis and Sp sprout. (**b**) A plot comparing the two quality indicators for each variable (variable = chemical shift identified as a determined metabolite) from a discriminant analysis (OPLS-DA): the variable importance in the projection (VIP) vs the variable reliability in the model (p[corr]). The separation between the two small regions (Sp = sprout; IE = inner epidermis) within the core of a single garlic clove is evident. With OPLS-DA quality-parameters: 1 predictive component and 1 orthogonal component, R^2^X = 0.747, R^2^Y = 0.955, Q^2^ = 0.828, and a p-value of the cross-validation = 0.027. Adapted with permission from [30]. Copyright (2018) American Chemical Society.

Capitalizing the merits of HR-µMAS, HR-µMAS NMR was successfully applied to comprehensive metabolic profiling of a food tissue [30]. Figure 5a shows the sampling of µg tissues with good spectral quality data (15-min acquisition with 0.03 ppm resolution), permitting localized profiling of the different anatomical regions of a garlic clove: skin, flesh, core-sprout and core-inner epidermis. More impressively, it was capable of carrying out a discriminative analysis between the two tiny regions (core-sprout and core-inner epidermis) owing to the µg-sampling by HR-µMAS (Figure 5b). This is impractical with HR-MAS because of the necessity of mg-sampling, highlighting the values of µMAS. Essentially, this study with HR-µMAS offers a firsthand account of µMAS to µg-analysis. 

## 3. Challenges in µMAS

### 3.1. Sample-Preparation 

Sample-preparation is a critical component in virtually all bioanalytical methods for acquiring representable data concerning the interest. Standardized experimental protocols are necessary and have been established in most NMR-based applications [22,31,32] to metabolomics. However, owing to the infancy of the μMAS development, currently, no robust protocols have been established. Sample-preparation may seem trivial at first glance, but it is, in fact, a demanding task for handling specimens—especially with semi-solids—at a microscopic level. One must consider the entire procedure clean and quick for preserving for the metabolic integrity. Envision the following procedure: (i) a rapid sample-extraction using a fine-needle syringe; (ii) a direct sample-filling into a μ-size rotor and followed by (iii) a quick rotor sealing. All these steps must be handled with dedicated tools (for example Figure 4b) under a stereoscope. Moreover, one of the many complications is that the specimens should be under a cold (or favorable) temperature during the procedure. 

Acknowledging the difficulty, capillary-sampling has been proposed for HR-MACS [25,26] and HR-μMAS [33]. This permits the preparation away from the rotor, facilitating the sample-filling by simple capillary-suction for the solution or capillary-punch for the tissue. However, the drawback is the loss of detection sensitivity with a reduced filling factor. Disposable μ-rotor (made of Kel-F) has also been considered for HR-μMAS [29]. Similar to that of the bio-insert with HR-MAS, it allows a mass preparation, but the fragility of the Kel-F rotor has hindered a rapid preparation. 

The necessity of using a rotor for sample spinning requires a sample-filling step in the preparation, which can be time-consuming and damaging to the sample. Currently, the most efficient methods are the use of micro-pipette for the solution and micro-sampling punch for tissue and occasionally abet with gentle centrifugation. The average time requires about 10–20 min for tissues; and the more challenging specimens are cells and small organisms. Indeed, improvement is necessary. This may require new devices, such as microfluidic, for easing the sample-filling; or even an entirely new design of the μMAS probe that can facilitate the entire preparation. For example, a probe (or stator) that can accommodate a spinning μ-needle, μ-punch tip or μ-pipette tip without the need of a sample rotor. This would indeed eliminate the strenuous task of sample filling.

### 3.2. Detection Sensitivity

Although μcoil improves the mass-sensitivity (i.e., SNR per unit mass) of microscopic samples to its maximum capacity, the overall sensitivity is still intrinsically inferior as compared to that of a large-mass detection – if available – with the standard HR-MAS. As an example, the sensitivity (in SNR per unit acquisition period) from HR-MACS with 12 *C. elegans* worm population is nearly 200-fold less than that of HR-MAS with a population of 1000+ [26]. This indeed limits the capacity in the analysis.

It is well-established that data acquisition at high magnetic field enhances the sensitivity (SNR ∝ $B_{0}^{3/2}$) (e.g., the use of 21.1 T for profiling a μg-tissue [23] and a single-organism [26]), but at a cost of increasing the susceptibility gradient effect (ΔS ∝ B_0_), and may lead to uncorrectable (i.e., by shimming) line-broadening. Increasing the signal-averaging could also enhance the sensitivity (SNR ∝ $\sqrt{\mathrm{scan}}$); however, it demands a considerable acquisition period that could contribute to sample degradation. Therefore, designing a μMAS study should be made with caution.

### 3.3. High-Throughput Analysis

The capability of high-throughput analysis–with the help of automated sampling and data acquisitions–has made NMR spectroscopy an efficient analytical tool in metabolomics. The challenge with µMAS is the limitation in sample-preparation and detection sensitivity described above. Each factor requires an extensive time as compared to the conventional HR-MAS: 10–20 minutes for sample-preparation and another 30–120 min for data acquisition. Indeed, these are the bottlenecks of µMAS to metabolomics and have prevented from the high-throughput analysis.

## 4. But Why µMAS?

A prime advantage of analyzing a microscopic specimen is the high level of homogeneity in the sample. This allows a candid characterization of the interest metabolic responses and helps to unfold the complexity of the biological variability. On the contrary, this information often conceals or is lost in the analysis of bulk size sample because of its high heterogeneity. Often it requires complex chemometric schemes for extracting the relevant information. One beneficial factor of µMAS is the invasiveness nature on the specimen with a minimal sample-manipulation.

In clinical science, microscopic sample analysis—as a complementary approach to advanced imaging techniques—could enhance the sensitivity of recognizing the modified metabolic patterns of the disease in tissue and may improve on the prognosis, diagnosis and screening. The possibility of firsthand monitoring of the perturbations in metabolic responses of the diseases could offer an effective and immediate personalized treatment. Moreover, since tissue excision (in the order of few mg) is currently a common practice in medicine for mg tissue analysis and is considered a non-invasive approach; with µMAS a sub-mg analysis could benefit the process by not only minimizing the invasiveness with μg excision (i.e., a signification factor for examining tissue such as human infants); but it could also reduce the necessity of large-scale surgical procedure.

In health science, the homogeneous data analysis by the microscopic-level investigation (i.e., sampling the uniform cellular tissue) could elevate the identification of the significant biomarkers of abnormality such as cancer in general [34], Alzheimer [35], and diabetes or other metabolic syndromes [36]. For example, in neuroscience, profiling the isolated neuron and astrocyte cells may help to reveal detailed insights into the fundamental of the intercellular metabolic cooperation between neurons and astrocytes for furthering in the understanding of brain metabolism [37].

In biological and ecological science, like in health science, to be able to analyze a single organism or unicellular organisms could simplify the annotation of the metabolic variant associated to the biological variability and differential susceptibility of the environment. On this basis, one could investigate the long-term harmful effects of pollutants on endangered species [38,39] by monitoring the changes in the metabolism of a single mosquito larvae due to pesticides [40], or a single amphipod for the exposure to toxic marine sediments [41]. These examples could benefit from µMAS microscopic analysis because of the non-destructive nature of the experiment, especially with small-sample diameter, which diminishes the centrifugation force exerted on a spinning sample.

In agricultural science, the analysis with µg samples could provide direct insights into the integration and regulation of plant metabolism: from a single seed to a grown plant. For examples, the germination process in a seed is complex and of great importance for the development of the plant seeds to form a new individual. To be able to characterize the metabolic coordination of the distinct µ-regions in a seed could help the development of germination [42] and essentially the plant growth. This could benefit from a spatiotemporal µMAS NMR analysis on a single seed.

A spatiotemporal µMAS analysis could also benefit to plant research. Environmentally adverse conditions such as changes in soil salinity [43] or water scarcity [44] are adverse circumstances that affect directly to plant roots and, consequently, to plant growth. Understanding the metabolic response to such events in localized regions of a root may elucidate the tolerant mechanisms in plants.

Similarly, in food science, fruits and vegetables in particular, could benefit from the homogeneity in sampling by µMAS, by supporting in the precise detection of bioactive compounds within different regions [45,46,47]. Thus, the microscopic-level analysis can be used to follow fruit conditions, such as the ripening process—for obtaining the optimal content of beneficial components.

## 5. Concluding Remarks

Since the first report of a µMAS NMR profiling in 2012, substantial advancements on µMAS methodology have been made, offering glimpses of a future potential NMR analysis of µg-scaled specimens to metabolomics. One example is the concept of clinical NMR spectroscopy proposed by Jeremy K. Nicholson and his team in 2011 [48]; however, a major hindrance is the analysis of mg tissue by HR-MAS. It gives highly heterogeneous information preventing an instantaneous identification of biomarkers. On the contrary, the homogeneous data from a µMAS analysis could offer a prompt feedback to the medical staffs for their diagnosis and prognosis.

Despite the advancement, there is no question that further improvements in detection sensitivity and sample-preparation are necessary to unlock the bottlenecks of µMAS analysis, and may render the possibility of clinical NMR spectroscopy. We are convinced that one-day µMAS NMR analysis would become a vital component in -omics research, but it would take a collaborative effort among the different disciplines with complementary expertise to develop a reliable µMAS NMR methodology for high-quality data acquisition with high-throughput analysis.
